# In Response to “De Novo KRAS G12C-Mutant SCLC: A Case Report”

**DOI:** 10.1016/j.jtocrr.2022.100418

**Published:** 2022-09-27

**Authors:** Margaret Moore, Yuyang Zhuo, Jeffrey D. Mandell, Stephen G. Gaffney, Jeffrey P. Townsend

**Affiliations:** Department of Biostatistics, Yale School of Public Health, New Haven, Connecticut; Program in Computational Biology and Bioinformatics, Yale University, New Haven, Connecticut; Department of Biostatistics, Yale School of Public Health, New Haven, Connecticut; Department of Biostatistics, Yale School of Public Health, New Haven, Connecticut; Program in Computational Biology and Bioinformatics, Yale University, New Haven, Connecticut; Department of Ecology and Evolutionary Biology, Yale University, New Haven, Connecticut

To the Editor:

Balbach et al.^1^ report SCLC with a KRAS G12C somatic mutation at 92.6% variant allele frequency (VAF). KRAS G12C is a substantial driver of NSCLC, for which the inhibitor sotorasib has been approved for second-line treatment. Balbach et al.^1^ consider whether their patient with SCLC would benefit from this treatment as there is no approved KRAS G12C-targeted therapy in SCLC. Given the well-established oncogenicity of KRAS G12 mutations, it is natural to expect KRAS G12C to play on oncogenic role in tumors where it is found. Nevertheless, G12C is so rare in SCLC that the alternative hypothesis—that the variant confers little proliferative advantage—should be considered. Even passenger mutations can achieve high VAF by chance. By calculating site-specific neutral cancer cell mutation rates in sequenced SCLC tumors, we can quantify the relative strength of selection driving prevalence of KRAS G12C in comparison to known SCLC drivers, gaining insight into the potential therapeutic efficacy of a KRAS G12C inhibitor.^2^

To quantify the tumorigenic effect of KRAS G12C mutation, we aggregated somatic variant calls from whole-exome sequencing of 1042 NSCLC tumors^3^ and from sequencing of 514 SCLC tumors (110 whole-genome sequencing, 139 whole-exome sequencing, and 265 panel sequencing of driver genes).^3^ The NSCLCs featured 132 G12 mutations versus eight in SCLC (13% versus 2%, two-sided Boschloo’s test, *P* = 6.7 × 10^−16^) and 54 G12C mutations versus two in SCLC (5% versus 0.4%, two-sided Boschloo’s test, *P* = 7.4 × 10^−8^). The lower prevalence in SCLC is suggestive of reduced oncogenic effect, but the difference could be explainable by differences in underlying KRAS G12 mutation rates between SCLC and NSCLC. Therefore, we calculated neutral mutation rates and quantified selection for KRAS G12C in each cancer type with cancereffectsizeR 2.6.4.^2^ In NSCLC, the scaled selection coefficient for the KRAS G12C variant was 1.4 × 10^4^, 23rd of 3272 recurrent variants. In SCLC, it was 1.2 × 10^3^—12-fold lower, 1120th of 1259. Lower effect indicates a lesser role in the growth and proliferation of SCLC than of NSCLC. Treatment with sotorasib may trend less beneficial for patients with KRAS G12C SCLC than for patients with KRAS G12C NSCLC.

Potential treatments may also be informed by the effect sizes of other variants in the patient which are quantified at higher oncogenic effect than KRAS G12C. For example, a point mutation of *B2M* was present at 88.4% VAF. A similar *B2M* loss-of-function mutation in SCLC exhibits sixfold higher effect than KRAS G12C—ranking 382nd at 6.9 × 10^3^. Prospects for immune checkpoint therapy should be tempered by the presence of *B2M* mutation, which has been linked to mismatch-repair deficiency in colon cancer and to acquired resistance to immune checkpoint inhibitors in melanoma^4^; alterations correlate with immunotherapy resistance and with tumor immune escape in lung cancer.^5^ In summary, considerations of the potential outcomes of off-label–targeted treatments should be informed by the tumor-type–specific cancer effect sizes of the targeted variants.

## CRediT Authorship Contribution Statement

**Margaret Moore**: Conceptualization, Investigation, Writing—original draft, Writing—review and editing, Project administration.

**Yuyang Zhuo**: Software, Formal analysis, Investigation, Data curation, Writing—review and editing.

**Jeffrey Mandell**: Methodology, Software, Validation, Formal analysis, Data curation, Writing—review and editing.

**Stephen Gaffney:** Validation, Data curation, Writing—review and editing, Supervision.

**Jeffrey Townsend:** Conceptualization, Methodology, Resources, Writing—original draft, Writing—review and editing, Supervision, Project administration.
